# Fluorescence-based method is more accurate than counting-based methods for plotting growth curves of adherent cells

**DOI:** 10.1186/s13104-020-4914-8

**Published:** 2020-02-04

**Authors:** Túlio Felipe Pereira, Gabriel Levin, Carlos DeOcesano-Pereira, Amanda Schiersner Caodaglio, André Fujita, Aldo Tonso, Mari Cleide Sogayar

**Affiliations:** 1 Cell and Molecular Therapy Center (NUCEL), Department of Internal Medicine, School of Medicine, University of São Paulo, Rua Pangaré, 100, Cidade Universitária, São Paulo, SP 05360-130 Brazil; 2 Department of Biochemistry, Chemistry Institute, University of São Paulo, São Paulo, SP Brazil; 3 Centre of Excellence in New Target Discovery (CENTD), Butantan Institute, São Paulo, SP Brazil; 4 Department of Computer Science, Institute of Mathematics and Statistics, University of São Paulo, São Paulo, SP Brazil; 5 Department of Chemical Engineering, Polytechnic School, University of São Paulo, São Paulo, SP Brazil

**Keywords:** Growth curves, Cell proliferation assessment, Doubling time determination, Fluorescence-based method

## Abstract

**Objective:**

Cell growth curves constitute one of the primary assays employed to analyze cell proliferation dynamics of in vitro cultured cells under specific culture conditions. From the cell growth curve, it is possible to assess the behavior of proliferating cells under different conditions, such as drug treatment and genomic editions. Traditionally, growth curves for adherent cells are obtained by seeding the cells in multiple-well plates and counting the total number of cells at different time points. Here, we compare this traditional method to the fluorescence-based method, which is based on the CFSE fluorescence decay over time.

**Results:**

The fluorescence-based method is not dependent on the determination of the total number of cells, but rather is approached by assessing the fluorescence of a sample of single cells from a cell population at different time points after plating. Therefore, this method is not biased due to either cell loss during harvesting or to the presence of cellular debris and cell clumps. Moreover, the fluorescence-based method displays lower variation among different measurements of the same time point, which increases the reliability on the determination of lag, log and stationary phase transitions.

## Introduction

The development of in vitro cell culture technique provided the establishment of a variety of cell lines from different organisms, developmental stages and pathologic conditions. Currently, questions posed in several biomedical sciences fields may be addressed using the outstanding platform of in vitro proliferating cells [1]. The most traditional assay to characterize in vitro cell proliferation is the cell growth curve [2–4]. The characterization of in vitro cell proliferation by plotting a cell growth curve may be assessed by different approaches. Incorporation of nucleoside-analogues is used to identify cells in the S phase of the cell cycle, such as tritiated thymidine (^3^H-TdR) and 5-Bromo-2′-deoxyuridine (BrdU). Proteins associated with the cell cycle, such as Ki-67, phosphorylated-histone H3 and proliferating cell nuclear antigen (PCNA) are also used as cell proliferation reporters. In addition, cytoplasmic proliferation dyes, such as carboxyfluorescein diacetate succinimidyl ester (CFSE) and the cell trace violet (ThermoFisher Scientific, Cambridge, MA) have been employed to track proliferating cells [5].

CFSE is a cell-permeant non-fluorescent pro-dye. Once the molecule is inside the cell, the acetate group is cleaved by cellular esterases and the resulting green fluorescent carboxyfluorescein molecule is no longer membrane permeable, unable to leave the cell. It binds to free amine groups through the succinimidyl ester group and generates covalent dye-protein conjugates. CFSE was employed as a cell tracker in 1994 to identify proliferating lymphocytes after a stimulus [6]. When stimulated, lymphocytes proliferate and each daughter cell receives half the CFSE content from the mother cell upon cytokinesis. CFSE has become a powerful tool in the Immunology field [7–9]. Later on, CFSE was used to analyze the interference of drugs in cell lines proliferation [9, 10] and their doubling time [11].

The most classical approach to generate growth curves is based on counting proliferating cells at different time points. Formerly, it was performed using Neubauer chamber, which is a tremendously laborious and variable task. Most recently, automated cell counters were developed to facilitate and accelerate the cell counting process. Furthermore, an alternative method analyze cell proliferation was developed based on fluorescence decay tracking [11, 14]. Here, we use samples of proliferating adherent cells to generate the growth curve by both counting- and fluorescence-based methods. The comparison of the results indicates that fluorescence-based method is more accurate because is not biased by technical drawbacks that interferes counting-based method.

## Main text

### Materials and methods

#### Cell culture

Human embryonic kidney cells (HEK 293 cell line, ATCC CLR-1573, Rockville, MD) were cultured in Dulbecco’s modified Eagle’s medium (DMEM; Gibco, MD, USA) supplemented with 10% fetal bovine serum (FBS; Vitrocell, São Paulo, Brazil). Cells were cultured at 37 °C under a humidified atmosphere containing 95% air/5% CO_2_.

#### Cells staining with CFSE

Cells were labeled using the CellTrace^TM^ CFSE Cell Proliferation Kit (Thermo Fisher Scientific, Waltham, MA, USA; C34554), according to the manufacturer’s instructions, with a few modifications. Cells (1 × 10^6^) were washed with PBSA (Phosphate- buffered saline without calcium and magnesium), ressuspended in CFSE solution in PBSA (5 µM CFSE—1 mL final volume) and incubated for 20 min at 37 °C on a side-to-side shaker. A volume (9 mL) of 10% FBS DMEM was then added and the cells were incubated for 5 min at 37 °C in a side-to-side shaker in order to allow free CFSE to bind to serum proteins and improve free CFSE elimination. Labeled cells were centrifuged, ressuspended in 10% FBS DMEM and seeded for the growth curve experiment.

#### Growth curves

CFSE stained cells (5 × 10^4^) were seeded onto 35 mm wells in 10% FBS DMEM, with culture medium change every other day. Triplicate wells were harvested by trypsinization at the indicated time points and the cells were fixed in 1 mL final volume of 3.7% formaldehyde. Growth curves were generated using four different approaches, namely: manual cell counting using the Neubauer Chamber, automatic cell counting using the Coulter Counter Analyzer (Beckman Coulter), automatic cell counting using the Accuri C6 Cytometer (BD Biosciences) and analysis of CFSE signal decay, also using the Accuri C6 Cytometer (BD Biosciences).

#### Automatic cell counting using the Accuri C6 Cytometer (BD Biosciences)

An end point acquisition stop was set at 100 µL for absolute cell counting. Cells were gated apart from debris in an SSC-A × FCS-A plot and the number of events were multiplied by 10 to yield the total number of cells per milliliter.

#### CFSE signal measurement using the Accuri C6 Cytometer (BD Biosciences)

Cells were gated apart from debris in a SSC-A × FCS-A plot. Single cells were then gated apart from the doublets and clumps in a FCS-H × FSC-A plot. An end point acquisition stop was set at 4000 events inside the single cells gate, from which the CFSE median fluorescence intensity (MFI) was determined. The MFI values were plotted as a function of time to analyze the kinetics of CFSE decay. Next, the inverse of MFI (values were raised to the power of − 1; MFI^−1^) was plotted as a function of time in order to change the plot from descendent exponential into an ascendant exponential.

#### Doubling time calculation

The cell specific growth rate (µ) was determined from the slope of the natural logarithm of cell count or MFI^−1^ as a function of time and doubling time (DT) using the formula [DT = ln 2/µ] [12].

#### Statistical analysis

Statistical analysis of the coefficient of variation of three replicates of the cell growth curves, determined by the different methods, was carried out by paired Wilcoxon tests. Analysis of CFSE MFI intensity and number of cells from time points 144 h to 168 h, in the presence or absence of 20 µg/mL Mitomycin C, was carried out using the t test.

### Results

**Fig. 1:**
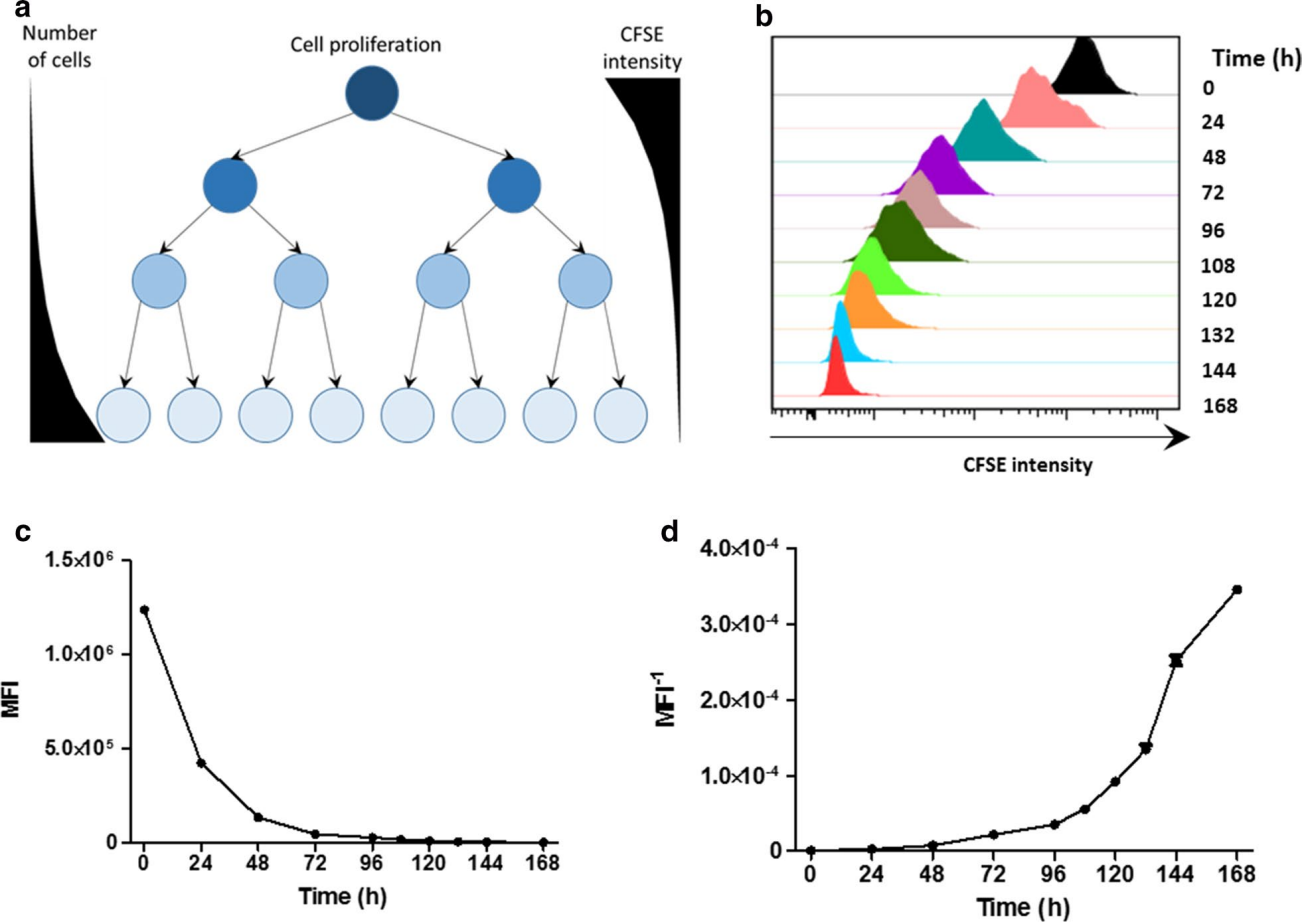
Analysis of CFSE signal decay upon cell proliferation. **a** CFSE passively diffuses into the cells and covalently binds to free amine residues. As cells divide, each daughter cell holds half of the CFSE content present in the mother cell. **b** HEK 293 cells were stained with CFSE and cultured for up to 7 days. Cells were harvested and fixed at different time points and the CFSE fluorescence signal was measured. Consecutive cell divisions lead to progressive CFSE signal decay in the cell population. The experiment was carried out with three technical replicates. **c** CFSE MFI as function of time reveals the exponential CFSE signal decay. **d** The inverse of CFSE MFI values (MFI^−1^) plotted as function of time renders the curve into a sigmoid, similar to the conventional growth curves

HEK 293 cells were stained with CFSE, seeded onto several wells and cultured for 7 days. Cells were harvested at different time points and the CFSE MFI was determined using the Accuri C6 Cytometer (BD Biosciences; Fig. 1b). The CFSE MFI from each time point was plotted as a function of time rendering an exponential descendent curve (Fig. 1c). Then, the inverse of CFSE MFI measurements were plotted as a function of time, transforming the descendent exponential plot into an ascendant plot and fitting very closely the conventional cell growth curve (Fig. 1d).

**Fig. 2:**
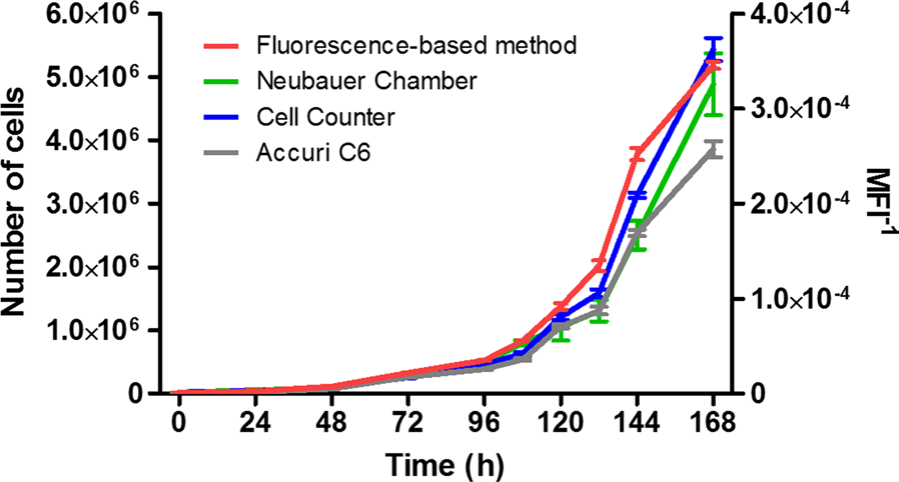
Comparison of HEK 293 growth curves plotted by counting-free fluorescence-based method and counting-based methods. HEK 293 cells were stained with CFSE and cultured for up to 7 days. Cells were harvested and fixed at different time points. The experiment was carried out with three technical replicates. The total number of cells (left Y axis) was determined at each time point using three different types of equipment, namely: Neubauer Chamber, Coulter Counter Analyser (Beckman Coulter) and Accuri C6 Cytometer (*BD* Biosciences). The CFSE MFI at each time point was determined using the Accuri C6 cytometer, and its inverse (MFI^−1^) was plotted in the right Y axis

Furthermore, the absolute number of cells in the same samples were determined using different types of equipment and plotted as counting-based cell growth curves. The counting-based curves were generated by using three different counting devices, namely: the Neubauer chamber, the Coulter Counter Analyzer Cell Counter (Beckman Coulter) and the Accuri C6 Cell Counter (BD Biosciences). The fluorescence-based curve was plotted together with the counting-based ones, confirming that the curves were similar (Fig. 2).

We quantitatively analyzed the results obtained using the different methods by comparing the doubling time values obtained from each of the curves. The fluorescence-based method delivered the doubling time 18 h 56 min, slightly lower than those calculated from the counting-based method (Neubauer chamber—20 h 41 min; Cell Counter—20 h 05 min and Accuri C6—20 h 16 min; Additional file 1: Table S1).

**Fig. 3:**
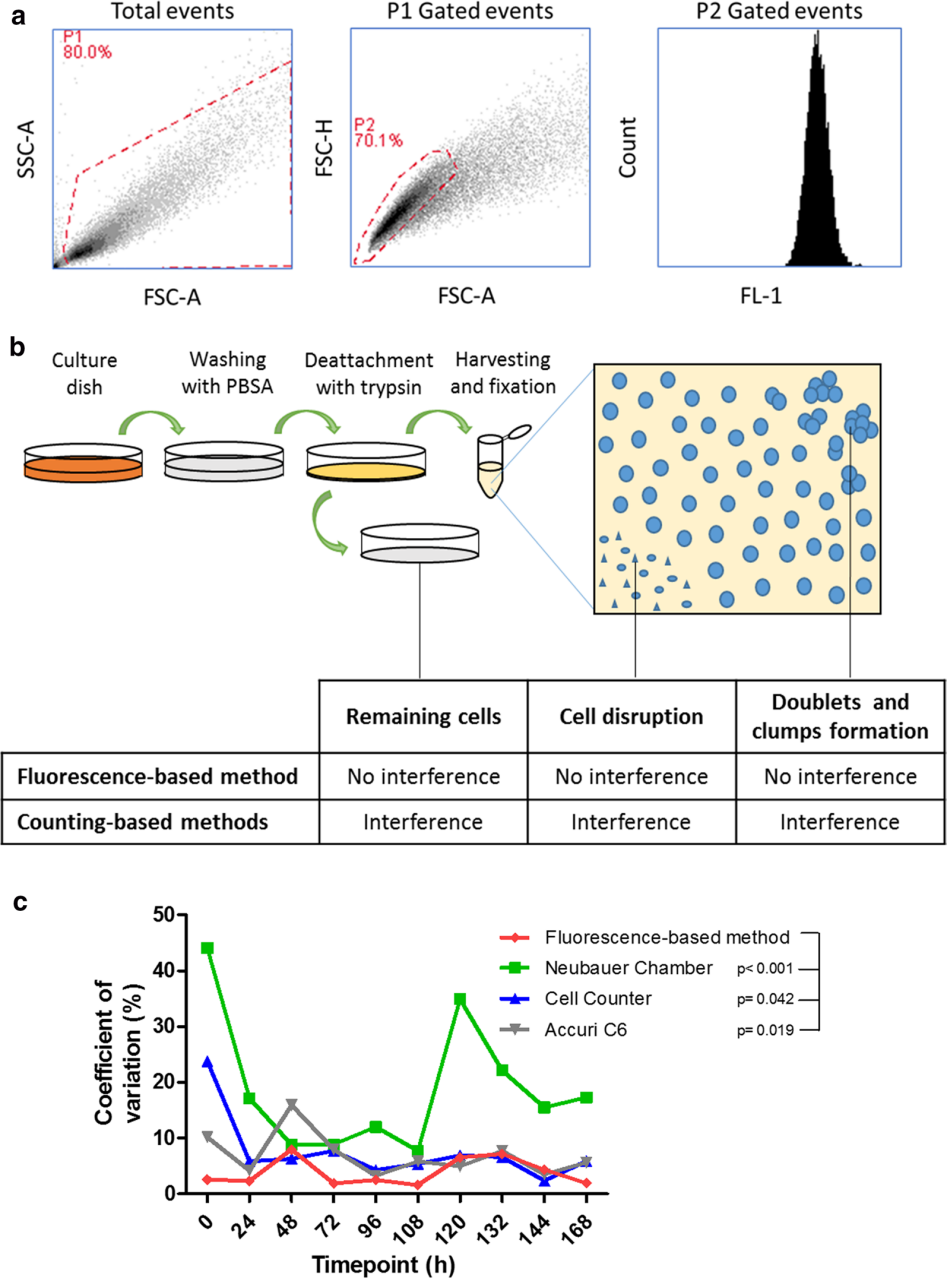
Variation among replicate measurements for each dataset. **a** Gating strategy for the determination of CFSE MFI for each time point. Cells were gated apart from debris in SSC-A × FCS-A plot. Considering only P1 gated events, single cells were gated apart from the doublets and clumps in FCS-H × FSC-A plot, and the CFSE MFI was measured considering only single cells. The analysis considered 4000 events into the single cells gate. **b** The presence of debris, doublet and clumps after cell harvesting and fixation interfere the final results of counting based methods, as well as those cells that remain in the plate after harvesting. On the other hand, these influences does not interfere on fluorescence-based method, which is based on the CFSE signal intensity of a small sample of single cells from each time point. **c** The coefficient of variation of three replicates for each time point. Statistical analysis was carried out by paired Wilcoxon test

The accuracy of cell growth curves generated by counting-based method depends on the precision in the determination of the total number of cells. On the other hand, this is not a requirement for fluorescence-based method, since the MFI of a small sample of single cells reveals the MFI of the whole population (Fig. 3a). The presence of cellular debris and cell clumps had no interference on MFI determination, as well as the number of cells left behind during cell harvesting. Nonetheless, these factors are important interferences on counting-based methods (Fig. 3b).

Furthermore, the values variation among technical replicates were lower in fluorescence-based method when compared to the counting one. This is quantitatively demonstrated by comparing the coefficient of variation of the triplicate measurements for each time point of the curve (Paired Wilcoxon test; Fig. 3c). This comparison also indicates the higher accuracy of fluorescence-based method.

Finally, we investigated the two last time points (144 and 168 h) to address the reliability on matching CFSE signal decay and cell proliferation. At these late time points, the CFSE has been maintained in conditions prone to spontaneous CFSE degradation for a long time (cytoplasmatic metabolism, 37 °C temperature). CFSE degradation regardless to cell division would abrogate the synchrony between fluorescence decay and cell proliferation. To address this question, the MFI of these last two time points were compared using Mitomycin C to halt cell proliferation. The number of cells did not increase upon Mitomycin C treatment and no CFSE signal decay was observed, confirming that CFSE decay matches to cell proliferation even after 7 days under cell culture conditions (Additional file 1: Figure S1). Since CFSE signal decays over time, this method may have limitations for longer-term studies, and this validation is requested for these cases.

### Discussion

The growth curve plot is the most common analysis to characterize in vitro cultured cells under different conditions, such as genomic manipulations [10, 14], presence of biomaterial [15] and treatment with chemical compounds [16]. Here we compared cell growth curves plotted by counting- and fluorescence-based methods.

Counting-based methods constitutes a laborious task, in addition to be influenced by cell loss during cells harvesting, which directly affects the total number of counted cells. Moreover, cellular debris and cell clumps increase the underestimation of total number of cells in cell-counting based growth curves. On the other hand, fluorescence-based method constitutes the usage of fluorescence cell tracers to stain cells (Fig. 1) and tracking proliferating cells by analysis of fluorescence decay [13]. As a result, the fluorescence of a cell population decreases as a function of time as cells proliferate, allowing this methodology to be employed to assess cell proliferation and to determine cell lines doubling time [11, 14]. This method is based on assessing the fluorescence signal of a sample of single cells from a cell population (Fig. 3a), therefore it is not influenced by cell loss during cell harvesting, cellular debris or cell clumps.

These interferences in counting-based method lead to an underestimation of the number of cells. It biases the cell growth curve towards a lower inclination (Fig. 2), which favors a longer doubling time (Additional file 1: Table S1). Three main interferences are detailed below (Fig. 3b).

First, the counting-based method is influenced by cell loss during cell harvesting from the culture plate. Cell growth curves usually start with a few cells in the first time points and a much higher number of cells at the last time points, reflecting a comprehensive representation of cell proliferation dynamics. The coefficient of variation among the three replicates in the first time points, in the case of the counting based methods, is much greater than that of fluorescence-based method, because any cell loss during harvesting has a great influence, considering the low number of cells present at these first time points (Fig. 3c). Losing cells during harvesting has no interference in the fluorescence method, considering that a small sample of harvested cells is sufficient to address the CFSE MFI of single cells at any time point. Second, cells which are disrupted into cellular debris are not taken into consideration in the counting-based methods, since they are out of the range of detection, underestimating the total number of cells. Third, whether or not the doublets and cell clumps are considered, the cell counting-based methods are biased towards underestimation of the total number of cells, especially in the last time points, when the cellular density is higher.

Furthermore, the lower variation among measurements of the same time points in fluorescence-based methods is another advantage in comparison to counting-based methods (Fig. 3c). The development of accurate techniques to increase the precision of cell growth curves is important to provide reliability in determination of Lag, Log, and Stationary phase transitions. Moreover, it is also crucial for characterization of cell lines used for production of recombinant proteins in the Biotechnology Industry. Moreover, cell tracers with different spectra may be used to stain different cell lines, which allows the analysis of several cell lines simultaneously [11]. Therefore, another advantage of fluorescence-based method is the possibility of analyzing co-cultured cells, which is not possible by counting-based methods.

We conclude that the generation of growth curves of adherent cells by fluorescence-based methods have three main advantages in comparison to counting-based methods. First, fluorescence-based method does not overestimate doubling time because it is not influenced by cell loss during harvesting, debris and clumps. Second, the variation among different measurements of the same time points is lower, which increases the reliability on Lag, Log and Stationary phase transition. Third, this method allows analysis of co-cultured cells.

### Limitations

- The fluorescence methods are more expensive because it requires both fluorescence dye and the cytometer equipment.
- The fluorescence-based method requests knowledge and training on cytometry to be carried out.

## Supplementary information

**Additional file 1: Table S1.** Doubling time comparison. **Figure S1.** Analysis of CFSE stability in the last two time points of the cell growth curve. Cell proliferation was stopped at time point 144 h using Mitomycin C to analyze proliferation-independent CFSE MFI decay. The experiment was carried out with three technical replicates. Statistical analysis was carried out by t test. **a** Mitomycin C treatment abrogates cell proliferation. **b** No CFSE MFI decay is observed in non-proliferating cells. **Table S2.** Raw cell counts data using the Accuri C6 Cytometer (*BD* Biosciences). **Table S3.** Raw cell counts data using the Neubauer chamber. **Table S4.** Raw cell counts data using the Coulter Counter Analyzer Cell Counter (Beckman Coulter). **Table S5.** Raw data from CFSE MFI measurements using the Accuri C6 Cytometer (*BD* Biosciences).

## Data Availability

The datasets used and/or analysed during the current study are available from the corresponding author on reasonable request.

## Abbreviations

3H-TdR: Tritiated thymidine
BrdU: 5-Bromo-2′-deoxyuridine
PCNA: Pproliferating cell nuclear antigen
CFSE: Carboxyfluorescein diacetate succinimidyl ester
DMEM: Dulbecco’s modified Eagle’s medium
FBS: Fetal bovine serum
PBSA: Phosphate-buffered saline without calcium and magnesium
MFI: Median fluorescence intensity
DT: Doubling time

## References

[1] Stacey G, Cohen IR, Lajtha A, Lambris JD, Paoletti R, Rezaei N (2012). Current developments in cell culture technology. Advances in experimental medicine and biology.

[2] Kantardjieff A, Zhou W (2013). Mammalian cell cultures for biologics manufacturing. Advances in biochemical engineering/biotechnology.

[3] Marx U (2012). Trends in cell culture technology. Advances in experimental medicine and biology.

[4] Freshney RI (2010). Culture of animal cells.

[5] Romar GA, Kupper TS, Divito SJ (2016). Research techniques made simple: techniques to assess cell proliferation. J Invest. Dermatol.

[6] Lyons AB, Parish CR (1994). Determination of lymphocyte division by flow cytometry. J Immunol Methods.

[7] Quah BJC, Parish CR (2012). New and improved methods for measuring lymphocyte proliferation in vitro and in vivo using CFSE-like fluorescent dyes. J Immunol Methods.

[8] Quah BJC, Warren HS, Parish CR (2007). Monitoring lymphocyte proliferation in vitro and in vivo with the intracellular fluorescent dye carboxyfluorescein diacetate succinimidyl ester. Nat Protoc.

[9] Rabah D, Grant S, Ma C, Conrad DH (2001). Synthesis bryostatin-1 specifically inhibits in vitro IgE bryostatin-1 specifically inhibits in vitro IgE synthesis 1. J Immunol.

[10] Chang WLW, Kirchoff V, Pari GS, Barry PA (2002). Replication of rhesus cytomegalovirus in life-expanded rhesus fibroblasts expressing human telomerase. J Virol Methods.

[11] Chung S, Kim S-H, Seo Y, Kim S-K, Lee JY (2017). Quantitative analysis of cell proliferation by a dye dilution assay: application to cell lines and cocultures. Cytom. Part A.

[12] Castilho L, Moraes A, Augusto E, Butler M. Animal cell technology—from biopharmaceuticals to gene therapy. 1st ed. Milton Park: Taylor & Francis; 2008.

[13] Begum J, Day W, Henderson C, Purewal S, Cerveira J, Summers H, Rees P, Davies D, Filby A (2013). A method for evaluating the use of fluorescent dyes to track proliferation in cell lines by dye dilution. Cytom Part A.

[14] Chaubey N, Ghosh SS (2015). Overexpression of granulocyte macrophage colony stimulating factor in breast cancer cells leads towards drug sensitization. Appl Biochem Biotechnol.

[15] Goldman EB, Zak A, Tenne R, Kartvelishvily E, Levin-Zaidman S, Neumann Y, Stiubea-Cohen R, Palmon A, Hovav A-H, Aframian DJ (2015). Biocompatibility of tungsten disulfide inorganic nanotubes and fullerene-like nanoparticles with salivary gland cells. Tissue Eng Part A.

[16] Jiang L, Yang N, Yuan X, Zou Y, Zhao F, Chen J, Wang M, Lu D (2014). Daucosterol promotes the proliferation of neural stem cells. J Steroid Biochem Mol Biol.

