# Supplementary material for: Fluorescence-based method is more accurate than counting-based methods for plotting growth curves of adherent cells
Source: BMC Res Notes. 2020 Feb 4;13:57. doi: 10.1186/s13104-020-4914-8 (PMC7001368; doi:10.1186/s13104-020-4914-8)
Supplement: Supplementary file 1 — Additional file 1: Table S1. Doubling time comparison. Figure S1. Analysis of CFSE stability in the last two time points of the cell growth curve. Cell proliferation was stopped at time point 144 h using Mitomycin C to analyze proliferation-independent CFSE MFI decay. The experiment was carried out with three technical replicates. Statistical analysis was carried out by t test. a Mitomycin C treatment abrogates cell proliferation. b No CFSE MFI decay is observed in non-proliferating cells. Table S2. Raw cell counts data using the Accuri C6 Cytometer (BD Biosciences). Table S3. Raw cell counts data using the Neubauer chamber. Table S4. Raw cell counts data using the Coulter Counter Analyzer Cell Counter (Beckman Coulter). Table S5. Raw data from CFSE MFI measurements using the Accuri C6 Cytometer (BD Biosciences). [file 13104_2020_4914_MOESM1_ESM.doc]

Additional file 1

**Additional file 1:** **Table S1.** Doubling time comparison.

| **Method** | **Doubling time** |
| --- | --- |
| **Fluorescence-based method** | 18h 56min |
| **Neubauer Chamber** | 20h 41min |
| **Cell Counter** | 20h 05min |
| **Accuri C6** | 20h 16min |


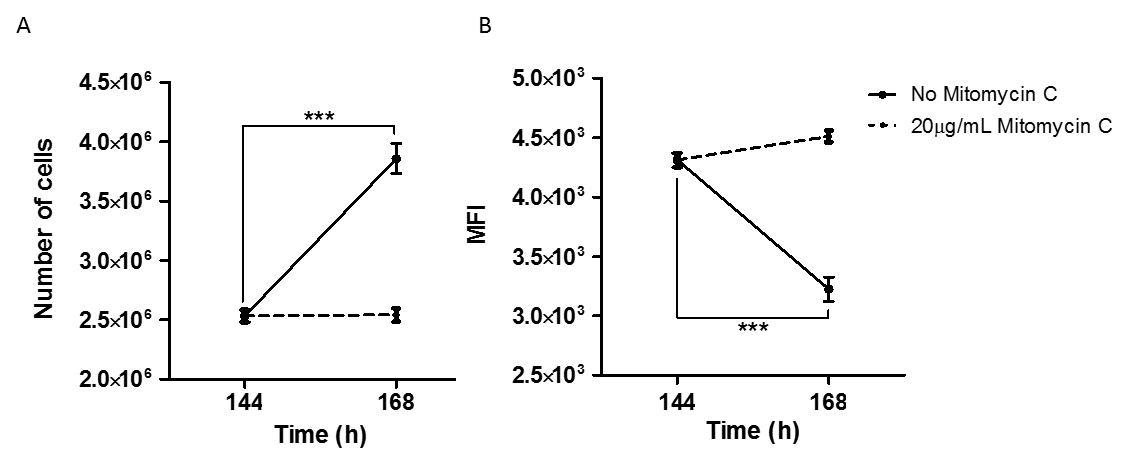


**Additional file 1: Figure S1. Analysis of CFSE stability in the last two time points of the cell growth curve.** Cell proliferation was stopped at time point 144h using Mitomycin C to analyze proliferation-independent CFSE MFI decay. The experiment was carried out with three technical replicates. Statistical analysis was carried out by t test. (A) Mitomycin C treatment abrogates cell proliferation. (B) No CFSE MFI decay is observed in non-proliferating cells.

**Additional file 1: Table S2.** Raw cell counts data using the Accuri C6 Cytometer (BD Biosciences).

| **Time (h)** | **Replicate 1** | **Replicate 2** | **Replicate 3** | **Mean** | **Standard Deviation** | **Coefficient of variation (%)** |
| --- | --- | --- | --- | --- | --- | --- |
| 0 | 1,542 | 1,563 | 1,294 | 1,466.3 | 149.6 | 10.2 |
| 24 | 2,803 | 3,047 | 2,949 | 2,933.0 | 122.8 | 4.2 |
| 48 | 7,782 | 6,605 | 9,098 | 7,828.3 | 1,247.1 | 15.9 |
| 72 | 24,055 | 26,690 | 28,205 | 26,316.7 | 2,100.0 | 8.0 |
| 96 | 38,045 | 37,597 | 39,960 | 38,534.0 | 1,255.1 | 3.3 |
| 108 | 51,494 | 52,554 | 57,402 | 53,816.7 | 3,149.9 | 5.9 |
| 120 | 106,249 | 101,220 | 111,833 | 106,434.0 | 5,308.9 | 5.0 |
| 132 | 122,405 | 128,901 | 142,290 | 131,198.7 | 10,139.7 | 7.7 |
| 144 | 256,655 | 243,015 | 259,874 | 253,181.3 | 8,950.2 | 3.5 |
| 168 | 368,472 | 378,051 | 410,115 | 385,546.0 | 21,809.8 | 5.7 |

**Note**: Cell counting using the Accuri C6 Cytometer was carried out with 100µL of each sample.

**Additional file 1: Table S3.** Raw cell counts data using the Neubauer chamber.

| **Time (h)** | **Replicate 1** | **Replicate 2** | **Replicate 3** | **Mean** | **Standard Deviation** | **Coefficient of variation (%)** |
| --- | --- | --- | --- | --- | --- | --- |
| 0 | 2.25 | 1 | 1.25 | 1.5 | 0.7 | 44.1 |
| 24 | 6 | 5.5 | 4.25 | 5.3 | 0.9 | 17.2 |
| 48 | 9.25 | 8.5 | 7.75 | 8.5 | 0.8 | 8.8 |
| 72 | 28.25 | 30.75 | 33.75 | 30.9 | 2.8 | 8.9 |
| 96 | 43.5 | 46 | 54.5 | 48.0 | 5.8 | 12.0 |
| 108 | 82.25 | 85.5 | 73.5 | 80.4 | 6.2 | 7.7 |
| 120 | 115 | 136 | 64.5 | 105.2 | 36.8 | 34.9 |
| 132 | 135 | 100 | 157.5 | 130.8 | 29.0 | 22.1 |
| 144 | 210 | 252.5 | 287.5 | 250.0 | 38.8 | 15.5 |
| 168 | 510 | 395 | 560 | 488.3 | 84.6 | 17.3 |

**Note**: The numbers are the average of four Neubauer chamber quadrants.

**Additional file 1: Table S4.** Raw cell counts data using the Coulter Counter Analyzer Cell Counter (Beckman Coulter).

| **Time (h)** | **Replicate 1** | **Replicate 2** | **Replicate 3** | **Mean** | **Standard Deviation** | **Coefficient of variation (%)** |
| --- | --- | --- | --- | --- | --- | --- |
| 0 | 19,600 | 16,800 | 12,000 | 16,133.3 | 3,843.6 | 23.8 |
| 24 | 42,400 | 47,600 | 44,400 | 44,800.0 | 2,623.0 | 5.9 |
| 48 | 106,800 | 98,600 | 94,400 | 99,933.3 | 6,306.6 | 6.3 |
| 72 | 257,600 | 280,400 | 240,400 | 259,466.7 | 20,065.2 | 7.7 |
| 96 | 450,000 | 486,000 | 452,800 | 462,933.3 | 20,025.3 | 4.3 |
| 108 | 614,800 | 669,600 | 607,200 | 630,533.3 | 34,045.5 | 5.4 |
| 120 | 1,308,000 | 1,153,000 | 1,174,000 | 1,211,666.7 | 84,085.3 | 6.9 |
| 132 | 1,504,000 | 1,556,000 | 1,706,000 | 1,588,666.7 | 104,887.2 | 6.6 |
| 144 | 3,108,000 | 3,067,000 | 3,212,000 | 3,129,000.0 | 74,746.2 | 2.4 |
| 168 | 5,120,000 | 5,758,000 | 5,416,000 | 5,431,333.3 | 319,276.3 | 5.9 |

**Note**: Number of cells per mililiter.

**Additional file 1:Table S5.** Raw data from CFSE MFI measurements using the Accuri C6 Cytometer (BD Biosciences).

| **Time (h)** | **Replicate 1** | **Replicate 2** | **Replicate 3** | **Mean** | **Standard Deviation** | **Coefficient of variation (%)** |
| --- | --- | --- | --- | --- | --- | --- |
| 0 | 1,272,412 | 1,212,068 | 1,222,749 | 1,235,742.9 | 32,202.0 | 2.6 |
| 24 | 428,813 | 411,320 | 427,511 | 422,548.0 | 9,745.2 | 2.3 |
| 48 | 122,389 | 141,198 | 140,890 | 134,825.9 | 10,771.5 | 8.0 |
| 72 | 44,798 | 46,260 | 46,366 | 45,807.9 | 876.6 | 1.9 |
| 96 | 28,317 | 29,340 | 27,947 | 28,534.5 | 721.6 | 2.5 |
| 108 | 17,837 | 17,831 | 18,338 | 18,002.0 | 290.6 | 1.6 |
| 120 | 10,163 | 11,582 | 11,057 | 10,934.3 | 717.6 | 6.6 |
| 132 | 7,844 | 7,674 | 6,841 | 7,453.0 | 536.6 | 7.2 |
| 144 | 3,852 | 3,902 | 4,175 | 3,976.3 | 173.6 | 4.4 |
| 168 | 2,955 | 2,846 | 2,885 | 2,895.2 | 55.6 | 1.9 |

**Note**: The MFI was measured from 4000 event from the single cells gate.
